# The roles of vicariance and isolation by distance in shaping biotic diversification across an ancient archipelago: evidence from a Seychelles caecilian amphibian

**DOI:** 10.1186/s12862-020-01673-w

**Published:** 2020-08-26

**Authors:** Simon T. Maddock, Ronald A. Nussbaum, Julia J. Day, Leigh Latta, Mark Miller, Debra L. Fisk, Mark Wilkinson, Sara Rocha, David J. Gower, Michael E. Pfrender

**Affiliations:** 1 Present address: Faculty of Science and Engineering, University of Wolverhampton, Wolverhampton, WV1 1LY UK; 2 Department of Life Sciences, The Natural History Museum, London, SW7 5BD UK; 3 Department of Genetics, Evolution and Environment, University College London, WC1E 6BT, London, UK; 4 Island Biodiversity and Conservation Centre, University of Seychelles, Mahé, Seychelles; 5 Research Museums Center, University of Michigan, Ann Arbor, MI 48108 USA; 6 Division of Natural Sciences and Mathematics, Lewis-Clark State College, Lewiston, ID 83501 USA; 7 Department of Biology, Utah State University, Logan, UT 84322 USA; 8 McPherson Eye Research Institute, University of Wisconsin, Madison, WI 53705 USA; 9 Biomedical Research Center (CINBIO), University of Vigo, Vigo, Spain; 10 Galicia Sur Health Research Institute, Vigo, Spain; 11 Department of Biological Sciences, University of Notre Dame, Notre Dame, Indiana, 46556 USA

**Keywords:** Adaptation, AFLPs, Biogeography, Caecilian, Evolution, Islands, Morphology

## Abstract

**Background:**

Island systems offer excellent opportunities for studying the evolutionary histories of species by virtue of their restricted size and easily identifiable barriers to gene flow. However, most studies investigating evolutionary patterns and processes shaping biotic diversification have focused on more recent (emergent) rather than ancient oceanic archipelagos. Here, we focus on the granitic islands of the Seychelles, which are unusual among island systems because they have been isolated for a long time and are home to a monophyletic radiation of caecilian amphibians that has been separated from its extant sister lineage for ca. 65–62 Ma. We selected the most widespread Seychelles caecilian species, *Hypogeophis rostratus*, to investigate intraspecific morphological and genetic (mitochondrial and nuclear) variation across the archipelago (782 samples from nine islands) to identify patterns and test processes that shaped their evolutionary history within the Seychelles.

**Results:**

Overall a signal of strong geographic structuring with distinct northern- and southern-island clusters were identified across all datasets. We suggest that these distinct groups have been isolated for ca. 1.26 Ma years without subsequent migration between them. Populations from the somewhat geographically isolated island of Frégate showed contrasting relationships to other islands based on genetic and morphological data, clustering alternatively with northern-island (genetic) and southern-island (morphological) populations.

**Conclusions:**

Although variation in *H. rostratus* across the Seychelles is explained more by isolation-by-distance than by adaptation, the genetic-morphological incongruence for affinities of Frégate *H. rostratus* might be caused by local adaptation over-riding the signal from their vicariant history. Our findings highlight the need of integrative approaches to investigate fine-scale geographic structuring to uncover underlying diversity and to better understand evolutionary processes on ancient, continental islands.

## BACKGROUND

Islands have had an important influence on the understanding of diversification and adaptive evolution since the landmark publication of “On The Origin of Species by Means of Natural Selection” [1] see [2]. These systems have been the focus of numerous evolutionary studies in part because they are particularly tractable for investigating the consequences of evolutionary processes such as random genetic drift, founder effects and local adaptation e.g. [3–7]. The majority of these studies have focused on relatively rapidly evolving radiations on emergent oceanic island groups, such as Caribbean *Anolis* lizards e.g. [8–11], Galápagos finches e.g. [12–14], and Hawaiian spiders e.g. [15]. In contrast, there have been few studies investigating the effects that these evolutionary processes might have on lineages evolving on ancient continental islands, which are characterized by much greater timescales and likely lower overall vagility of resident taxa. To increase our understanding of evolutionary patterns and processes on ancient continental islands, we focus on a caecilian amphibian species endemic to the granitic Seychelles.

The Seychelles Archipelago comprises approximately 115 islands in the western Indian Ocean. The 41 granitic islands lie ca. 1500 km east of mainland East Africa (centered around 55°30′ E and 4°30′ S) and provide a rare example of oceanic islands supporting multiple lineages of insular amphibians. The remaining 74 islands are of more recent coralline origin and are devoid of amphibians. The granitic islands are of continental origin [16], and are mountain-top remnants of the largely submerged Seychelles Microcontinent (“Seychellea” of [17]) that once formed part of the supercontinent Gondwanaland. These ancient remnants were isolated by the separation from Africa of a landmass (Indigascar of [18]) consisting of Madagascar, Seychelles and India [19–24]; with the Seychelles becoming fully separated approximately 65–62 Ma [24, 25]. Much of the Seychelles Microcontinent, comprising the exposed (ca. 250 km^2^ [26]) and submerged granitic Seychelles, currently lies submerged at a mean depth of 55 m and encompasses an area of 129,650 km^2^ [27]. Throughout their history, the individual granitic islands of the archipelago have been variously connected and sundered by eustatic sea level changes, and the Seychelles Microcontinent was likely maximally emergent as recently as 10 kya [28–32]. The granitic Seychelles are considered the emergent parts of an isolated continental block [33].

Despite the key attributes of the Seychelles system such as its continental origin, truly oceanic setting, and mosaic of ancient (including Gondwanan relictual) lineages and more recent overwater arrivals that make it attractive for studying evolutionary processes, the Seychelles biota remains poorly studied at the intraspecific level. Greater use of molecular techniques has underpinned a recent increase in the number of studies of phylogeographic patterns in the Seychelles [34–48]. These studies have documented some common e.g. [40, 49] and some different [36, 50] spatial patterns of genetic diversity, including ‘cryptic’ diversity within some taxa. In many taxa within the Seychelles (lizards [39, 44–46, 51, 52], frogs [40, 48] and crabs [47]) there is a broadly similar pattern of geographic structuring, with distinct northern vs. southern island group lineages. Generating comparative data for additional taxa, especially for phylogenetically and ecophenotypically disparate lineages and for taxa that have been resident in the Seychelles for varying durations, might allow common patterns of genetic and phenotypic variation to be identified. These analyses should enable more powerful tests of hypothesized biotic and abiotic causes of evolution within this ancient continental island system.

Seychelles is home to eight currently recognized species of caecilian amphibians (Gymnophiona) in three genera [53–55], belonging to a single, monophyletic radiation which has likely been isolated from confamilals since the Seychelles separated from India [25]. The presence of endemic amphibians (especially caecilians) on oceanic islands is highly unusual, and supports the hypothesis that the Seychelles Microcontinent has been at least partly emergent throughout the Cenozoic Era (see [56]). Here, we focus on *Hypogeophis rostratus* [57] because it is the most widespread endemic amphibian in the Seychelles, occurring on 10 of the granitic islands [56]. Phylogenetic relationships among Seychelles caecilians remain incompletely resolved, including identification of the closest extant relative of *H. rostratus* [38, 55]. However, analyses of a previously published dataset [25] using the same methods reported in that study found that, applying nine different calibration strategies, estimated (mean) ages of splits among sampled extant species of *Hypogeophis* and *Grandisonia* are in the range of 16.4–27.9 Ma (95% posterior density ranges 11.0–37.5 Ma) (D. San Mauro, *pers. comm.*). *Hypogeophis rostratus* resides in a variety of habitats and elevations and is often found in high abundance in human altered habitat such as coconut plantations and gardens (*pers. obs.*). Although it can be seen in streams and pools, typically at night, *H. rostratus* is largely terrestrial and soil dwelling as adults, lays terrestrial eggs, has direct development [58, 59] and exhibits geographically structured morphological variation [60–62]. Despite the large range and seemingly adaptable nature of *H. rostratus*, the species is assumed to be less vagile (at least among islands) than many other Seychelles species that have been investigated phylogeographically (e.g. lizards) because it is largely fossorial and because of the osmotic properties of amphibian skin e.g. [63, 64], which make it intolerant of salt water [65].

Patterns of morphological diversity do not always represent phylogeny because factors such as local ecological adaptation and phenotypic plasticity can play a role in shaping phenotypic variation e.g. [66], including at the intraspecific level [67–69]. To understand evolutionary patterns and processes it can therefore be beneficial to analyze both genetic and morphological data. Few biologists have examined morphological or molecular intraspecific variation in caecilians across the Seychelles or elsewhere. Recent intraspecific studies of caecilians have generally been based on small sample sizes and/or have made only brief comments on morphology [70–73]. The three largest studies of intraspecific genetic and morphological variation in caecilians published to date are dominated by molecular data. Gower et al. [74] reported little variation and slight geographic structuring of mtDNA variation within the Indian ichthyophiid *Ichthyophis bombayensis* across a large area of peninsular India. Stoelting et al. [75] reported substantial, geographically structured morphological and mtDNA variation within the dermophiid *Schistometopum thomense* on the small Gulf of Guinea island of São Tomé. Wang et al. [76] reported substantial, geographically structured mtDNA and nuclear microsatellite variation in the ichthyophiid *I. bannanicus* in Indochina.

Here we present results of analyses of morphological and genetic variation in *H. rostratus* from across its range, representing the largest, most detailed examination of caecilian intraspecific variation to date. The primary questions we address are: what are the main patterns of genetic and phenotypic variation within *H. rostratus* across the Seychelles archipelago, and what processes are responsible for them?

## Results

### Molecular data

#### Mitochondrial and nuclear DNA sequence data

**Fig. 1:**
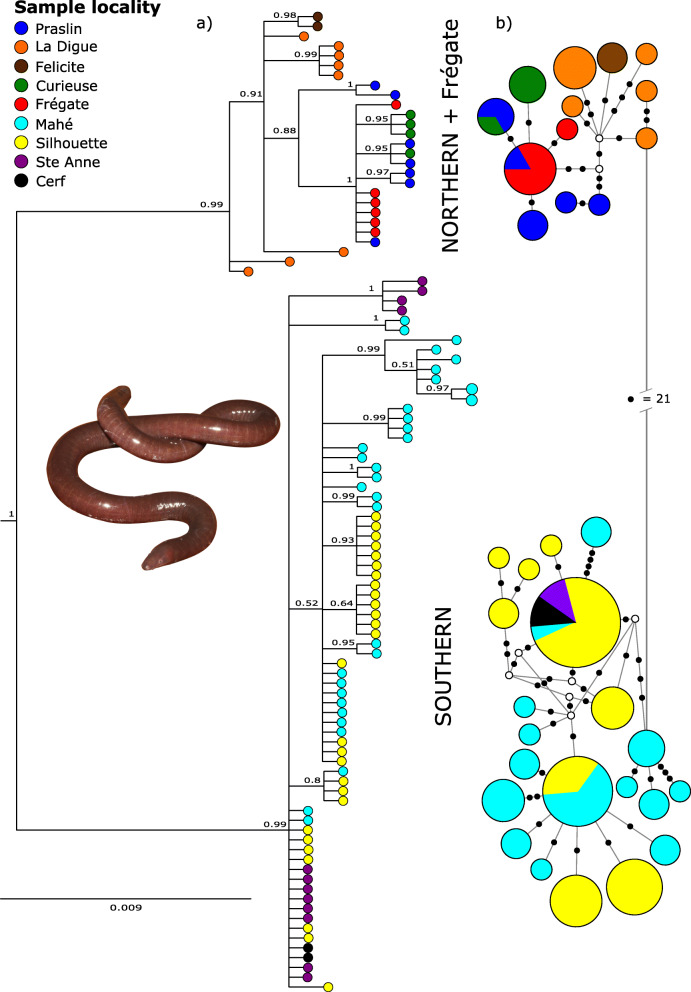
Evolutionary relationships of *Hypogeophis rostratus* inferred from *cytb* data: **a**) Bayesian inference majority rule consensus tree. BI posterior probabilities > 0.5 are presented on the branches; **b**) Median-Joining haplotype network. Size of the haplotypes are indicative of number of individuals in the haplotype. Small black circles refer to mutational steps between haplotypes and open circles represent extinct or unsampled haplotypes. a & b) Colors on tips of the BI tree and within the haplotypes refer to sample island of origin as referred to in the key (and are the same as those used in Fig. 2). Depicted image of *H. rostratus* is from Mahé

Based on variation in 700 bp of the mitochondrial encoded cytochrome b gene (*cytb)* we identified 34 unique haplotypes among 100 *H. rostratus* individuals sampled from nine islands. C*ytb* data was partitioned into codon positions (CP) based on the results of PartitionFinder [77] analysis, and for the Bayesian inference (BI) analyses run through MrBayes [78] using the substitution models HKY + I (CP1), HKY (CP2) and GTR (CP3). Phylogenetic analyses using BI revealed a strongly supported, basal split between specimens from the northern islands + Frégate and specimens from the southern islands (Fig. 1a). The clustering pattern in the *cytb* phylogeny is congruent with the *cytb* haplotype network (Fig. 1b). The sharing of some haplotypes between islands could be indicative of shared ancestry (with recent vicariance not yet reflected in the molecular markers) and/or of some migration having occurred in the recent past between islands.

Northern + Frégate island vs. southern island individuals are separated by *p*-distances of 3.2–4.8% (between group mean distance = 3.7%; net between group distance = 3.4%: Table 1). The maximum amount of genetic variation within the two main lineages is greater within the southern island group (*p*-distances up to 1.6%: between individuals from the island of Mahé) than within the northern + Frégate group (*p*-distances up to 1%: between individuals from La Digue and Praslin).

**Table 1:** Nucleotide diversity

	*cytb*	*bdnf*	*pomc*	*brev5*	*rost5*
All islands	0.019	0.001	0.006	0.008	0.004
Northern group	0.006	0.000	0.004	0.008	0.002
Southern group	0.005	0.001	0.005	0.000	0.004
Praslin	0.004	0.000	0.004	0.000	0.002
La Digue	0.004	0.000	–	0.004	0.002
Curieuse	0.001	0.000	0.005	0.020	0.004
Felicite	0.000	0.001	0.003	0.000	0.002
Frégate	0.000	0.000	0.003	0.003	0.002
Mahe	0.006	0.001	0.003	0.000	0.003
Silhouette	0.004	0.000	0.007	0.000	0.004
Ste. Anne	0.003	0.001	0.005	0.000	0.004
Cerf	0.000	0.001	0.003	0.000	0.002
North vs south	0.039	0.000	0.007	0.013	0.004
*D*	0.240	−1.869	−0.475	0.021	−0.411

Nucleotide diversity of genetic sequence data for 100 (100 *cytb*, 25 *bdnf*, 19 *pomc*, 26 *brev5*, 25 *rost5*) *Hypogeophis rostratus* individuals, with mean *p*-distance between northern and southern island groups and Tajima *D’*s

**Fig. 2:**
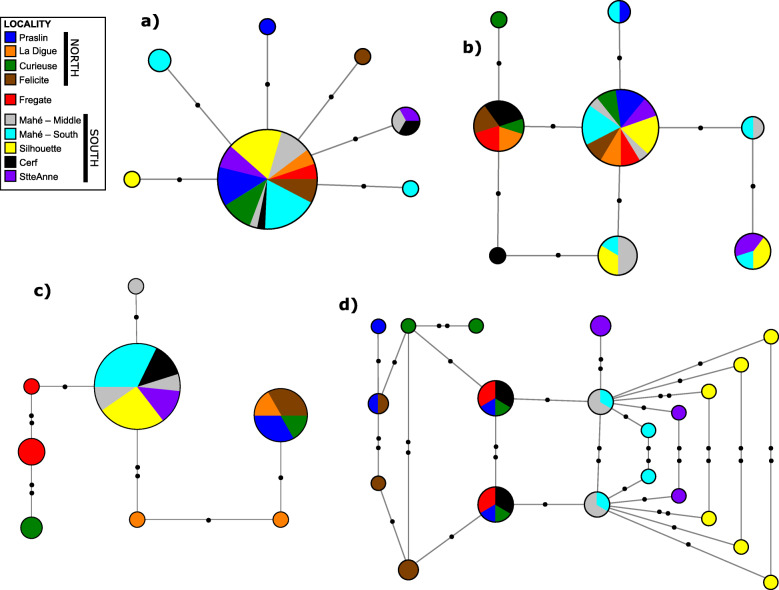
Haplotype network for *Hypogeophis rostratus* inferred from nuclear DNA: **a**) *bdnf*, **b**) *rost5*, **c**) *brev5,* **d**) *pomc*. Size of the haplotypes are indicative of number of individuals in the haplotype and colors indicate island population as referred to in the key. Small black circles refer to mutational steps between haplotypes and open circles represent extinct or unsampled haplotypes

Four nuclear loci—pro-opiomelanocortin (*pomc*), brain-derived neurotrophic factor (*bdnf*), and two anonymous nuclear markers (*brev5* and *rost5* [79];)—were sequenced for a subset of the samples sequenced for *cytb* (Additional file 2). As expected, mtDNA nucleotide diversity is greater than for nuDNA (see Table 1). No nuclear haplotypes for *H. rostratus* are restricted to single islands or larger within-island regions except for the samples from Frégate for *brev5* (Fig. 2). For *brev5* there is a widespread northern island haplotype and a widespread southern island haplotype. Specimens from the eastern island of Frégate and one specimen from the northern island of Curieuse are more similar to southern island individuals. For *pomc* there are main northern + Frégate and southern-island clusters, though samples from the southern island of Cerf share alleles with two widespread northern island haplotypes; a pattern also observed in the widespread northern island haplotype for *rost5*. For *bdnf* and *rost5,* allele sharing is extensive and there are no clear distinctions between specimens from northern- and southern- island groups. For *pomc,* specimens from the islands of Mahé, Silhouette and St. Anne all have unique haplotypes.

Inference under the isolation with migration (IM) model [80] supports that no migration is occurring between the northern island + Frégate and southern island groups (Additional File 3). StarBEAST analyses estimate that the northern island + Frégate and southern island groups diverged approximately 1.26 Ma [95% HPD: 0.49–2 Ma] for the Yule tree prior (1.11 Ma [95% HPD: 0.37–1.84 Ma] for the Birth Death tree prior, and 1.27 Ma [95% HPD: 0.51–2.03 Ma] under a Coalescent tree prior).

#### AFLP analyses

AFLP genetic diversity varies considerably among islands. Using Nei’s [81] unbiased heterozygosity, diversity estimates ranged from ~ 0.19 (Mahé) to ~ 0.04 (Praslin) (Table 2). Analyses of AFLP data identified considerable genetic structuring among islands. Estimates of θ were large, and 95% confidence limits of θ did not overlap with zero (θ = 0.3574; upper CI = 0.4548, lower CI = 0.2670).

**Table 2:** Genetic diversity measures for *Hypogeophis rostratus*

	Island						
Island	Mahe	Silhouette	Ste. Anne	La Digue	Frégate	Praslin	Curieuse
Mahe	***0.1897***	0.0262	0.0349	0.2724	0.1606	0.4350	0.3786
Silhoutte	0.00026	***0.1757***	0.0514	0.2800	0.1814	0.4291	0.3749
Ste. Anne	0.00243	0.00188	***0.1652***	0.2783	0.1857	0.4326	0.3590
La Digue	0.03237	0.03247	0.03333	***0.0963***	0.1113	0.1916	0.1851
Frégate	0.03728	0.03704	0.03790	0.00495	***0.1181***	0.4291	0.3749
Praslin	0.03728	0.03704	0.03790	0.00495	0.00571	***0.0417***	0.0611
Curieuse	0.03776	0.03752	0.03838	0.00543	0.00048	0.00619	***0.0628***

Genetic diversity measures for *Hypogeophis rostratus* within and between islands. Values on the diagonal are within-island average dissimilarity based on AFLP markers for 274 individuals. Values above the diagonal are Nei’s (1978) genetic distance (d) based on AFLPs, and values below the diagonal are the net number of substitutions per site (D_a_) between islands

**Fig. 3:**
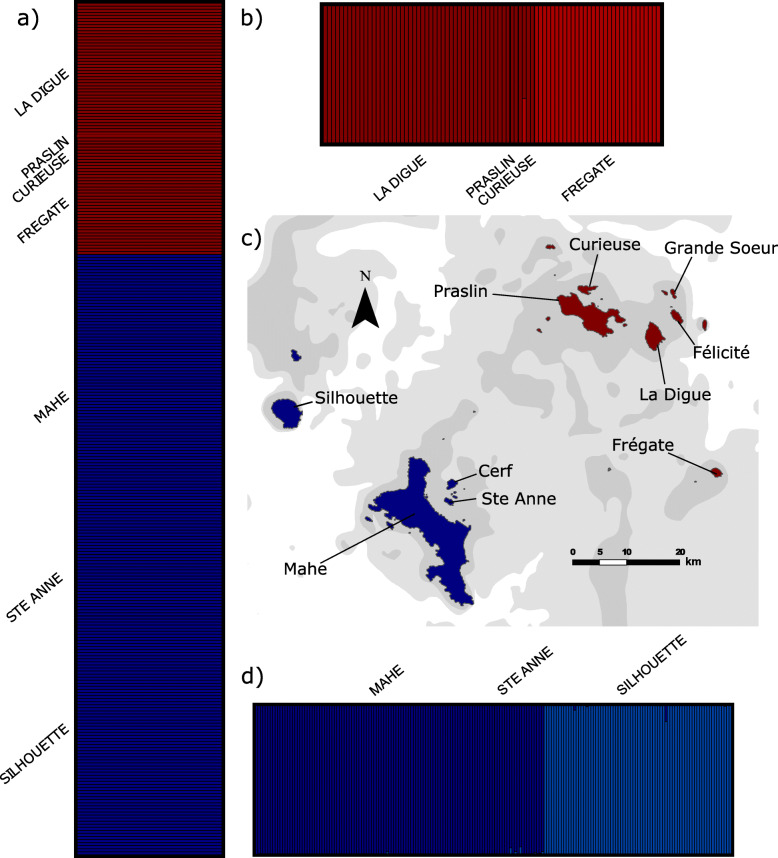
Island sampling localities and population clustering of *Hypogeophis rostratus* based on STRUCTURE analyses. AFLP clustering in: **a**) all specimens, **b**) the northern-island cluster identified from the all specimen analysis, **d**) the southern-island cluster identified from the all specimen analysis, **c**) map of Seychelles islands sampled during this study; dark grey contours = − 30 m/bpsl, light grey contours = − 50 m/bpsl

**Fig. 4:**
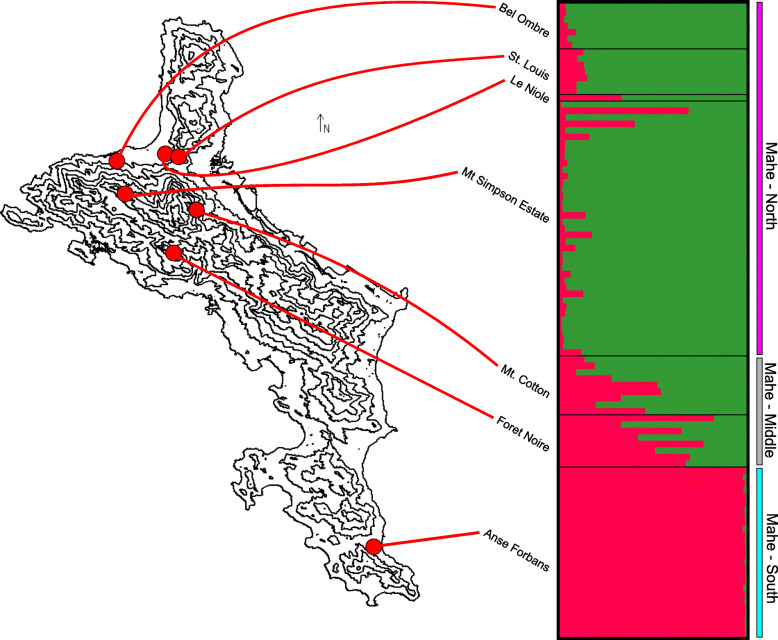
Intra-Mahé AFLP clustering of *Hypogeophis rostratus* samples from STRUCTURE analyses. Contour lines on map are at 100 m elevational intervals. Referred populations to the northern, middle and southern groups are indicated to the right of the STRUCTURE plot

In all STRUCTURE [82] analyses the optimal clustering of individuals was *K* = 2. Clusters reflect a northern + Frégate vs. southern island group split (Fig. 3). Variation within the northern + Frégate island group is further divided into specimens from the northern islands (Praslin, Curieuse and La Digue) and Frégate (Fig. 3b), with one individual from Curieuse reflecting admixture between the two clusters. Within the southern-island group there is an east-west geographic subdivision of genetic variation (*K* = 2), with individuals from Silhouette and individuals from the closer islands of Mahé and Sainte Anne forming separate clusters (Fig. 3c). Some individuals from each island show small levels of admixture. Within Mahé additional structuring was found (Fig. 4), with a separation between specimens from the most northerly (Bel Ombre, Le Niole, St. Louis, Mt Simpson Estate) and most southerly (Anse Forbans) sampled localities showing very little admixture. The somewhat intermediate (although still northerly) samples (Mt. Coton and Foret Noire) have shared alleles with both the northernmost and southernmost populations, in a clear geographic gradient.

UPGMA and PCA analyses further indicate that the relatively high levels of AFLP structure are explained primarily by the presence of two island groups (Additional file 1), the maximally supported southern islands group (samples from Mahé, Silhouette, Sainte Anne), and a much less strongly (60% bootstrap) supported northern islands (Praslin, Curieuse, La Digue) + Frégate group. Within the northern island + Frégate group there is a strongly supported (92% bootstrap) group comprising samples from Praslin, Curieuse, and La Digue (to the exclusion of Frégate). In the PCA plot (Additional file 1) there is substantial overlap among samples from the southern islands and among samples from the northern islands; the Frégate island population is completely distinguishable. Consistent with our UPGMA dendrogram, estimates of θ_ST_ obtained from these two main island groups (southern and northern + Frégate) are similar to overall levels of θ obtained when individual islands were treated independently (θ_ST_ for island groups = 0.3650; upper CI = 0.4666, lower CI = 0.2563). Ordination analyses provide qualitative support for patterns produced in the UPGMA dendrogram. Plots of the first two principal coordinates reiterated the differentiation of northern and southern island groups, and further illustrated the relatively loose alliance of individuals from Frégate with representatives of the northern island group (Additional file 1).

### Phenotypic data

#### Univariate analyses

**Fig. 5:**
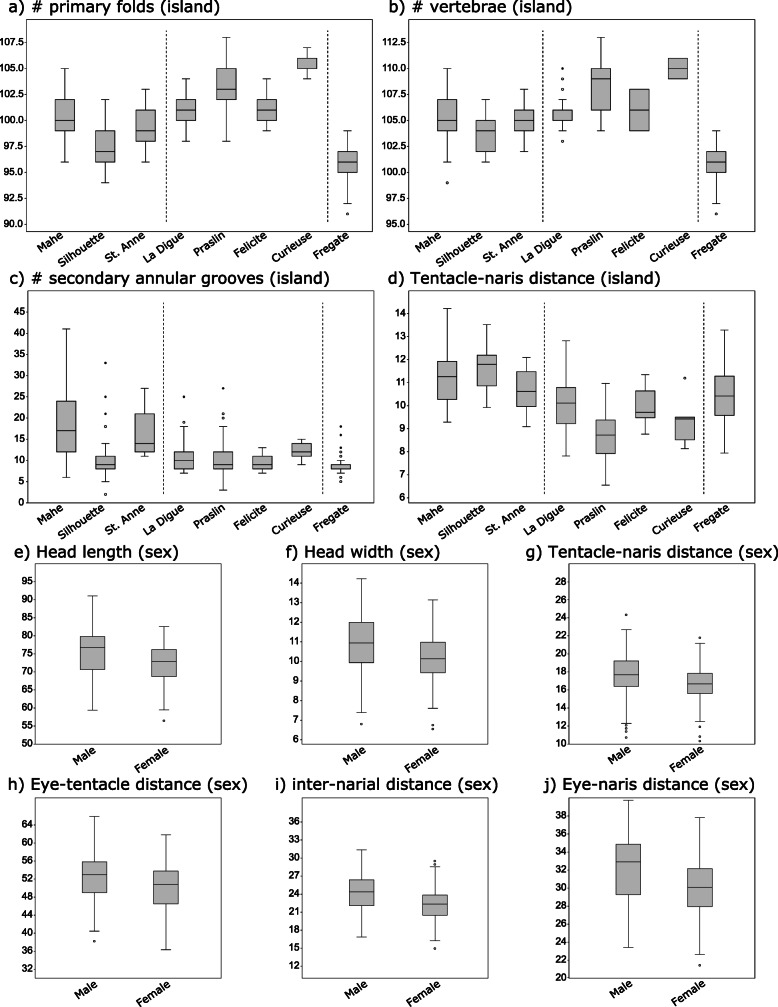
Boxplots of morphological data for *Hypogeophis rostratus,* depicting similarity among samples from different islands (**a**-**d**) and sex (**e**-**j**). Differences between islands in: **a**) number of primary folds, **b**) number of vertebrae, **c**) number of secondary annular grooves, **d**) TN. Sex comparison: **e**) HL, **f**) HW, **g**) TN, **h**) ET, **i**) IN, **j**) EN. The number of individuals used in boxplots were as follows: Mahé *n* = 51 males & 55 females, Silhouette *n* = 26 males & 15 females, Sainte Anne *n* = 8 males & 8 females, Frégate *n* = 18 males & 21 females, Praslin *n* = 23 males & 18 females, La Digue *n* = 30 males & 16 females, Curieuse *n* = 5 males, Félicité *n* = 9 males & 11 females

Body width and head trait dimensions are strongly sexually dimorphic in *H. rostratus* (Fig. 5). Females have wider bodies than males regardless of island of origin. In general, females have smaller adjusted mean values for head length and width, and shorter IO (see Methods for description of morphological measurement abbreviations), IN, EN, ET, and TN distances. The notable exception is females from Curieuse, which have larger adjusted mean values for head length and width, and longer IO, EN, ET, and TN distances. This difference is reflected in the significant interaction between sex and island of origin for all but one trait (IN distance) for head morphology.

Numbers of folds, scales and vertebrae (PF, VF, SF, VERT, SR, and PFS) vary significantly only with island of origin (Fig. 5; Additional file 1), although a significant interaction for vertebrae suggests that variation in this character is also somewhat dependent upon the sex of the individual. There are no distinct patterns in the differences for number of folds, folds with scales, scale rows, or vertebrae among islands.

#### Multivariate analyses

**Fig. 6:**
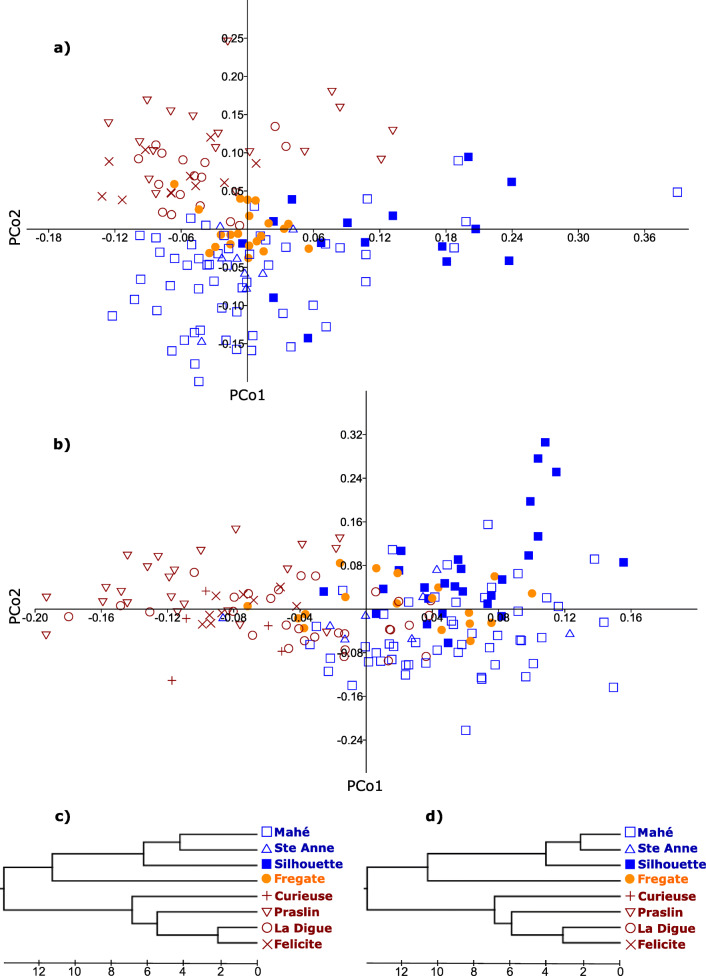
Multivariate analyses of *Hypogeophis rostratus* morphological data: **a**) PCoA plot of females, **b**) PCoA plot of males. UPGMA dendrograms based on Malhanobis’ D between all island pairs: **c**) males, **d**) females. Colors and symbols used in the PCoA plots, match those used in the UPGMA dendrograms. The same individuals were used as in Fig. 5

The PCoA plots (Fig. 6) for both males and females show little overlap between southern and northern island samples, and much more overlap and scatter for samples from individual islands within each of these two main groups. In this multivariate analysis of morphological traits, the Frégate samples overlap much more extensively with the southern island group samples than the northern island group. The PCA plots (Additional file 1) generally agree with the PCoA plots in terms of the similarity of samples from the different islands. For males, the first two principal components account for 92% of the variation in the data, with the first principal component (PC1) explaining 86% of the total variance. Factor loadings for PC1 were high and positive for head length and width (0.64 and 0.47, respectively) and moderately positive for other head traits (range: 0.11–0.35). Factor loadings for PC2 were high and positive for HW (0.74), moderately positive for IO (0.26) and BW (0.11), high and negative for HL (− 0.61) and low and negative (− 0.02 to − 0.08) for other head measures. For females, the first two principal components account for 89% of the variability in the data, with PC1 explaining 82% of the total. Similar to the pattern in males, factor loadings for PC1 were high and positive for head length (0.6) and width (0.53), and other head trait dimensions (range: 0.1–0.36). Factor loadings for PC2 were high and positive for HW (0.72), moderately positive for BW (0.2) and IO (0.24), and moderately negative for HL, EN, and ET (− 0.26–-0.46).

UPGMA dendrograms based on Mahalanobis distances among islands for males and females have identical branching patterns and differ only in branch length (Fig. 6). This analysis recovers two groups of islands separating the northern and southern islands, supporting the findings from the mtDNA and AFLP analyses. However, a notable difference to the genetic data is the placement of the Frégate samples, which are joined by a long branch to the group of southern islands (rather than clustering with the northern islands).

#### Phenotypic subdivision

The mean level of island group (northern island + Frégate, and southern island groups) subdivision for all morphological characters was 0.18 (P_ST_) in both males and females. Levels of phenotypic subdivision were high for VERT and PF in both males and females (males: 0.46 and 0.47, respectively; females: 0.52 and 0.48, respectively). Values for all remaining traits for males and females were between 0.09 and 0.24.

Although there is substantial evidence of sexual dimorphism in *H. rostratus,* as demonstrated by the ANCOVA analysis, there is no evidence that morphological variation among islands differs by sex. Mahalanobis distance between islands is highly correlated in males and females (Mantel test; r = 0.9, *p* = 0.001) (Additional file 1). A linear regression of P_ST_ for traits in females versus males shows a high correlation between the amount of island subdivision in the two sexes (r = 0.98, *p* < 0.001) (Additional file 1). The slope of the least-squared regression line is not significantly different from one (lower CI = 0.80; upper CI = 1.02) and the intercept not significantly different from zero (lower CI = − 0.01; upper CI = 0.03), indicating that male-female traits have evolved in a similar manner.

#### Combined data set analyses

Simple Mantel tests of distance matrix associations revealed significant correlations between all of the datasets apart from between mtDNA vs. female morphometric data and between male morphometric data vs. geographic distance (Table 3). These relationships are indicative of isolation-by-distance.

**Table 3:** Mantel and partial Mantel tests results for variation in *Hypogeophis rostratus*

	mtDNA		AFLP	
	*R*	*p*-value	*R*	*p*-value
**Simple Mantel**				
Genetic x Geographical	0.737	0.03	0.818	< 0.01
Genetic x Morphometric (♀)	0.356	n.s.	0.596	0.01
Genetic x Morphometric (♂)	0.561	0.01	0.534	0.02
Morphometric (♀) x Geographic	0.463	0.03		
Morphometric (♂) x Geographic	0.406	n.s.		
**Partial Mantel**				
Genetic x Geographic | Morphometric (♀)	0.691	0.04	0.761	< 0.01
Genetic x Geographic | Morphometric (♂)	0.673	0.02	0.778	< 0.01
Genetic x Morphometric (♀) | Geographic	0.024	n.s.	0.425	0.03
Genetic x Morphometric (♂) | Geographic	0.423	n.s.	0.384	0.02
Morphometric (♀) x Geographic | Genetic	0.319	n.s.	−0.051	n.s.
Morphometric (♂) x Geographic | Genetic	−0.013	n.s.	−0.063	n.s.

n.s. indicates a non-significant result for *p*-values

The partial Mantel test results indicate a significant association between genetic (mtDNA and AFLP) and geographic distance while controlling for morphology (male and female). There is a marginally significant association between AFLP and morphological (male and female) data while controlling for geography (Table 3).

The correlations between Nei’s d (AFLP) and D_a_ (mtDNA) between islands was *r* = 0.81 (*p* = 0.24). The relationships between molecular genetic divergence at AFLP markers and Mahalanobis distance were significant for males (*r* = 0.49, *p* = 0.024) and marginally significant for females (*r* = 0.43, *p* = 0.052). The correlations between the mtDNA based D_a_ and Mahalanobis distances for males and females were positive but not significant (males: *r* = 0.25, *p* = 0.134; females: *r* = 0.18, *p* = 0.147).

## Discussion

Genetic and morphological data for *Hypogeophis rostratus* generally support the presence of northern island (Praslin, La Digue, Curieuse, Felicite) and southern island (Mahé, St. Anne, Silhouette, Cerf) lineages. Samples from the most easterly island, Frégate, are morphologically distinct and evidence for their affinities to either the northern or southern island group differs for morphology and genetics. The sequence data suggest that no gene flow has been occurring between the two main mtDNA lineages—northern + Frégate and the southern island populations—in the recent past but that the two lineages have a Pleistocene divergence. Geographic expansions and subdivisions would have been facilitated by cyclically oscillating sea levels during this time [32]. Taken together with the AFLP data, the sharing of alleles among samples from different islands, regardless of mtDNA lineage, in the nuclear sequence data is likely indicative of ancestral polymorphism and incomplete lineage sorting rather than recent dispersals between islands.

The spatial structuring of phenotypic and genetic variation within *H. rostratus* (especially the partitioning into northern- and southern-island groups) is broadly similar to that observed within Seychelles lizards [39, 44–46, 51, 52], frogs [40, 48] and crabs [47]. This spatial (and temporal) pattern is likely caused by rising sea levels sundering formerly widespread populations and/or IBD across Seychelles taxa. The extreme ecomorphological disparity among different taxa with divergent northern and southern island lineages, some of which should not be as osmotically challenged as caecilians (i.e. lizards), shows that geographic distance and marine barriers have played a major role in shaping the evolutionary history of the Seychelles biota.

That the molecular tree supports a northern + Frégate island group, whereas morphology does not, could be explained by local ecological adaptation acting on the morphology of *H. rostratus* on Frégate. The small size of Frégate (ca. 2 km × 1 km), and the relatively low genetic variation observed in *H. rostratus* there, could be explained by genetic drift, founder effects and/or by local adaptation generating selective sweeps e.g. [3–7]. Adaptation could be a plausible explanation for the incongruence given that Frégate differs from both the cooler, wetter southern islands and the drier, warmer (and palm dominated) northern islands. However, further interpretation is complicated by the typically human modified nature of most habitats across the granitic Seychelles, and a lack of precise data on the native vegetation of Frégate and of detailed climate records. Genetic drift, particularly as a result of founder effects, is common in island systems, especially when new populations are formed either via colonization or isolation e.g. [83] and is a potential explanation of allelic fixation and reduced variation.

Association between the different datasets (Table 3) strongly supports the general patterns of genetic and morphological variation being explained by isolation-by-distance (IBD) rather than isolation-by-adaptation (IBA) within *H. rostratus*. This observation matches similar interpretations for Seychelles lizards [42–46], though formal tests have not been performed for these other taxa to address this. Some support exists for IBA in the *H. rostratus* AFLP dataset but application of a more stringent significance threshold (*P* = 0.001) has been recommended due to the high Type I errors in partial Mantel tests [84], which would deem the results obtained here non-significant. IBD and the marine barriers are almost certainly jointly responsible for the geographic patterns of variation observed, which are likely caused predominantly by vicariance rather than dispersal.

There is substantial, spatially structured intraspecific genetic variation within *H. rostratus* on the largest island of the Seychelles archipelago, Mahé. The genetic ‘break’ between a northernmost and more southerly group on Mahé lies among the high peaks (to a maximum of 905 m) within the Morne Seychellois National Park. We have encountered *H. rostratus* from sea level to 670 m, and so it seems unlikely that the genetic subdivision was caused by a high-elevation barrier to gene flow (there is no obvious extant barrier), and this is somewhat supported by the observation that *H. rostratus* from directly south of Morne Seychellois (from Foret Noire and Mt. Coton) share alleles with individuals from localities to both the north and south. It seems more likely that the observed genetic subdivision is the result of IBD (possibly amplified by other environmental gradients) rather than purely or mostly by elevation. Due to a lack of sampling on Mahé it is not clear whether a continual or staggered gradient would be observed throughout the island. Although there are no records of *H. rostratus* from many parts of the island, this is likely due to a lack of targeted surveys rather than an absence of the species from these areas because the species seems to cope well with human induced habitat modification. Comparative data for intraspecific genetic structuring within Mahé is not available for other taxa but see [46].

Of the largest previous studies investigating molecular (and in some cases morphological) intraspecific geographic variation in caecilians, all but one [74] found substantial genetic diversity [75, 76]. Both Stoelting et al. [75] and Wang et al. [76] suggested that genetic variation is associated with historic or present geographic barriers to gene flow rather than continuous, progressive IBD. In contrast, the marine barrier separating Seychelles islands coupled with IBD seem to be responsible for the spatially structured variation observed within *H. rostratus*.

We have uncovered substantial, spatially structured, intraspecific diversity associated with geographic barriers, based on the largest study of intraspecific geno- and phenotypic variation of a caecilian species to date. The effects that demography and vicariance have on caecilian species had not previously been addressed in such detail. The findings here suggest that some caecilian species might be more capable of dispersing (e.g. *H. rostratus* between geographically close islands) than would have been expected, due to the predominantly fossorial nature of the Order. Our results provide a platform for future research into how populations of organisms with osmotically sensitive skin, distributed across ancient island archipelagos, are structured within their natural environments. The findings also substantially improve our understanding of biotic distribution, diversity and diversification across the Seychelles.

### Taxonomy

*Hypogeophis rostratus* is morphologically distinct from congenerics and can be distinguished from other *Hypogeophis,* among many other features, by being much larger (all other species are < 120 mm in maximum length vs. > 400 mm), in having many more vertebrae (> 95 vertebrae vs. < 80) and in having scales and secondary annular grooves only on the posteriormost part of the body [54, 55]. Analyses of 16 s rRNA (the only marker for which data are available for all *Hypogeophis* species and for both main lineages of *H. rostratus*) confirms the distinctiveness of *H. rostratus* from other *Hypogeophis* (*p*-distance ca. 0.06% between northern and southern island lineages and a minimum of 8.5% between the genetically closest *Hypogeophis* (*H. montanus*). The morphology and genetic evidence (genetic distance within *H. rostratus* is two orders of magnitude lower than the minimum interspecific distance) provides strong support that the two main *H. rostratus* lineages are representative of infraspecific variation and not (taxonomically) associated with the three other currently recognized species of the genus.

Four subspecies have been formally described for *H. rostratus*: *H. r. rostratus* Parker, 1958 [60]*, H. r. guentheri* (Boulenger, 1882) [85]*, H. r. praslini* Parker, 1958 [60] and *H. r. lionneti* Taylor, 1969 [62]. *Hypogeophis R. rostratus* and *H. r. praslini* were described from types from the southwestern island of Mahé and northern island of Praslin, respectively. The holotype of *H. r. guentheri* was reported originally to be from Zanzibar (where no caecilians are known to occur), but Parker [60] suggested that it was likely instead to be from the Seychelles island of Frégate based on its low number of vertebrae. The holotype of *H. r. lionneti* is from an unspecified locality within the Seychelles. These subspecies have been ignored in the scientific literature since Taylor [61], including in conservation assessments [86].

Our data support the recognition of at least two distinct evolutionary significant units for *H. rostratus*. These conform to a northern lineage, potentially referable to *H. r. praslini* and a southern lineage potentially referable to *H. r. rostratus*. However, the incongruence of the affinities of specimens from Frégate with the northern (genetic) or southern (morphology) populations would prevent compelling assignment of Frégate *H. rostratus* to either of these two subspecies. The lack of reliable locality data for the types of *H. r. lionneti* and *H. r. guentheri* and the absence of discrete diagnostic characters for the island populations further complicates the situation. We recommend that no subspecies should currently be recognized for *H. rostratus,* pending a broader genomic sampling that allows us to better assess the levels of differentiation versus gene-flow across these islands. However, there is an argument for treating at least the northern island, southern island and Frégate populations as distinct conservation management units in order to preserve diversity. If marine barriers are maintained long into the future then the subunits identified here could be considered incipient species.

## Conclusions

Genetic and morphological data support a geographically structured northern island and southern island clade within the Seychelles caecilian *Hypogeophis rostratus*. The eastern island of Frégate is nested within the northern island clade in all molecular analyses, but conversely occupies a more intermediate position between the two clades based on the morphological data. It is likely that a combination of IBD and vicariance has shaped the geographic structure observed within the species, with a primary split between the populations estimated at ca. 1.2 MA [95% HPD: 0.45–1.94 MA], with no migration occurring between them, despite multiple low stands in sea level. The data support the recognition of a single but variable species.

## Methods

### Data collection

#### Sampling and DNA isolation

Typically, caecilians are considered difficult to find due to their mostly burrowing nature [87], such that substantial, dedicated fieldwork can be required to collect suitable numbers of samples for phylogeographic projects. This difficulty in sampling is perhaps one reason that very few studies of caecilian molecular ecology have been undertaken [74–76]. *Hypogeophis rostratus* were collected on nine granitic islands in the Republic of the Seychelles between 1981 and 1991, and between 2013 and 2015: Mahé, Sainte Anne, Cerf, Silhouette, Frégate, La Digue, Praslin, Curieuse, and Félicité (Fig. 3). Our sampling covers the known distribution of the species [56] with the exception of the small island of Grande Soeur. We included a total of 782 individuals in the various components of this study (Additional file 2). The specimens collected between 1984 and 1991 for molecular analyses (mt- & nuDNA sequences and AFLPs) were either returned live to the lab, subsequently euthanized, and liver and muscle tissues snap frozen and stored at − 80C at University of Michigan Museum of Zoology; or they were collected in the field and snap frozen. Additional tissue samples were collected between 2013 and 2015 for molecular analyses (mt- and nuDNA sequences) and were processed in the field or were non-lethally sampled (buccal swabs and/or annular pocket biopsies, following [88]) and stored in 100% EtOH. Specimens used for morphological analyses (collected between 1984 and 1991) were fixed in 10% buffered formalin and stored in 65–70% EtOH. Samples utilized in this study are represented by vouchers accessioned to the permanent collections of the Natural History Museum, London (BMNH, issued with SM field codes) and the University of Michigan Museum of Zoology (UMMZ) (Additional file 2). Genomic DNA from 312 individuals of *Hypogeophis rostratus* from nine islands was obtained from tissue by a standard [89] proteinase *K* digestion with either a phenol/chloroform/isoamyl alcohol extraction or a Qiagen DNeasy Blood and Tissue kit [see 88 for non-lethal DNA extraction protocols].

#### DNA amplification for sanger sequencing

The polymerase chain reaction (PCR) was used to amplify a portion of the mtDNA cytochrome *b* (*cytb*) gene from 100 individuals sampled from nine islands (Additional file 1) and four nuclear loci for 26 individuals from seven islands; samples for sequencing were semi-randomly selected in order to obtain geographic coverage. The nuclear loci consisted of portions of two protein coding genes (pro-opiomelanocortin (*pomc*) and brain-derived neurotrophic factor (*bdnf*)) and two anonymous nuclear markers *(brev5* and *rost5*) [79]. See Additional file 1 for primer, DNA amplification, sequencing and sequence editing details.

#### Amplified fragment length polymorphism (AFLP) data generation

AFLP data were used to assess nuclear genetic variation among sampled populations of *H. rostratus*. AFLP marker profiles were generated for a total of 274 individuals from seven islands (all of these seven islands were among those sampled in the mitochondrial and nuclear DNA sequence datasets: Additional file 2), collected between 1984 and 1991, using the protocol described by Vos et al. [90] with modifications described in Mock and Miller [91]. In the second selective amplification step, the following four primer combinations were used to generate multilocus DNA fingerprints: EcoACG/MseAGA, EcoACG/MseACT, EcoAGG/MseATC, EcoAGG/MseAGA. After the second selective amplification, PCR products were run on an ABI 3100 automated DNA sequencer (Applied Biosystems, Inc.) using a Rox400 internal size standard. Repeatability was checked by duplicating ca. 15% of samples. Following sequencer runs, the presence or absence of individual AFLP marker phenotypes were visualized and scored using Genographer [92]. Markers were scored if they were polymorphic (95% criterion) and could be scored unambiguously across the dataset. Scoring was performed without reference to sample or population identity. The final AFLP dataset included data from 49 polymorphic loci.

#### Phenotypic traits

Morphological data were generated for 506 individuals of *H. rostratus* from eight islands (including all islands that were sampled for mitochondrial and nuclear DNA sequence data: see Additional file 1). Only individuals collected between 1984 and 1991, that were in a good preservation state, were examined for morphology. The sex of 461 of the specimens was determined by direct examination of gonads (females = 231, males = 230). We examined 5 males and 4 females from Curieuse, 9 males and 11 females from Félicité, 17 males and 21 females from Frégate, 30 males and 15 females from La Digue, 98 males and 115 females from Mahé, 37 males and 39 females from Praslin, 2 males and 2 females from Sainte Anne, and 26 males and 17 females from Silhouette. A ruler was used to record total length to the nearest 1.0 mm. Dial calipers were used for all other measurements. Body width was measured to the nearest 1.0 mm, all remaining measurements to the nearest 0.1 mm. The morphological measures recorded were: total length (TL), body width at midbody (BW), head length measured dorsally from tip of snout to first groove of first collar (HL), head width at level of corner of mouth (HW), inter-ocular distance between medial borders of eyes (IO), inter-narial distance between medial borders of nares (IN), eye-naris distance from anterior margin of eye to posterior margin of naris (EN), eye-tentacle distance from anterior corner of eye to midpoint of tentacular aperture (ET), tentacle-naris distance from midpoint of tentacular aperture to posterior margin of naris (TN), number of primary annular folds (grooves) after second collar (PF), number of primary annular folds interrupted by the vent (VF), number of primary annuli bearing partial or complete secondary annular folds (SF), number of secondary folds that completely encircle the body (CSF), total number of vertebrae counted from x-ray plates (VERT), number of overlapping rows of scales in a posterior primary fold counted in the dorsal region (SR), and number of primary folds containing scales (PWS) determined using the method described by Wilkinson et al. [93].

### Data analyses

#### Mitochondrial and nuclear DNA sequence analyses

We inferred relationships among individuals by constructing haplotype networks and by inferring phylogenetic trees. Individual alleles for diploid nuclear sequences were reconstructed using PHASE v.2.1.1 [94]. Input files for PHASE were produced using seqPHASE [95]. PHASE was executed three times for each locus, at a random starting seed, for 1000 iterations, a 10 thinning interval, and 100 burn-in. Each run was examined for mean frequency concordance, and the run that was most similar to zero selected for further analysis. Sites with heterozygous probabilities of ≥0.7 were considered to be correctly called by PHASE, others being coded as IUPAC ambiguities. Haplotype networks for *cytb* and nuclear sequence data were constructed using the median-joining algorithm [96] as implemented in the software NETWORK v.4.611 (fluxus-engineering.com). Uncorrected pairwise (*p*-) molecular genetic distances and Tajima D values were estimated using MEGA X [97].

Phylogenetic trees were inferred for *cytb* using Bayesian inference (BI) methods. PartitionFinder v.2.1.1 [77] was used to identify the best-fitting partitioning strategy and models of sequence evolution. MrBayes v.3.2.2 [78] was used to infer the BI tree for *cytb*, sampling every 10,000 generations over 10^6^ generations with one cold and three heated chains. Tracer v.1.7 [98] was used to check that chain convergence and good mixing occurred for all parameters and that all effective sample size (ESS) values were > 200. Convergence was also investigated by assessing that the potential scale reduction factor (PSRF) was close to 1.0 for all parameters, that the average standard deviation of split frequencies was below 0.01 and that log probability was within a relatively stable range and not still increasing. The first 10% of trees were discarded as burn-in. *Cytb* sequence data for a congeneric endemic Seychelles caecilian, *H. brevis,* were obtained from GenBank (HQ444110), and used as a closely related outgroup [38] to root phylogenetic trees. BI analyses were performed on the CIPRES Science Gateway v.3.1 [99].

Based on results from the initial molecular analyses, the Isolation-with-Migration (IM) model [100] as implemented in IMa2 [80, 101] was used to estimate population sizes, migration and time since divergence between the two major *H. rostratus* clades found ((Praslin, La Digue, Felicite, Curieuse, Frégate) and (Mahé, Silhouette, Cerf, St Anne)). The null hypothesis is that, since divergence, there has been no gene flow between the two (northern and southern island-group) lineages because of a saltwater barrier separating the two groups [28–32]. Initially, the mitochondrial *cytb* gene was used in the analyses (as its mutation rate would allow us the estimate of the parameters in meaningful demographic units) but after multiple independent runs, double peaks were observed in several parameters, which were likely a consequence of reciprocal monophyly between the two groups for *cytb* data. Final IMa analyses therefore were carried out with only the nuclear data. No molecular rate was provided for the nuclear data because of a lack of comparative evolutionary rates for these markers. The IS model was used for the nuclear loci. Posterior density curves were inspected for clear peak estimates and appropriate prior distributions boundaries. Priors were defined based on summary statistics and adjusted following initial runs. The final analysis was run five times with different starting seeds and checked for consistency between runs.

Time estimates for the split between the northern- vs southern-island lineages were made using *BEAST in BEAST2, defining each clade as a “species” [102] for all of the sequence data (mt- and nuDNA). We estimated divergence dates based on an evolutionary rate of approximately 1% sequence divergence per million years with a lognormal distribution and a standard deviation of 0.0027 for *cytb,* following other studies of amphibians and reptiles [45, 103]. Nuclear rates were estimated relative to *cytb*. PartitionFinder v.2.1.1 [77] was again used to identify the best-fitting models of sequence evolution based, this time on each locus. Some of these were later replaced by their immediate less complex most similar model, when they failed to converge. The Yule Model prior was used as the tree-prior because we had no evidence of extinction in the population, and because choice of prior is unlikely to impact estimated divergence dates significantly [104]. As a test of time estimate robustness we also ran analyses using the Birth Death Model and the Coalescent (constant population size) priors. A log normal relaxed clock was used as the clock-prior for the *cytb* partition, and strict clock priors were used for the nuclear partitions (according to the coefficient of variation obtained during preliminary runs, a strict clock was considered appropriate to use). Analyses were run for 10^6^ generations, with sampling every 10,000 generations. Tracer was used to investigate that good mixing was occurring between all parameters and that ESS values were > 200 as per previous analyses.

#### AFLP analyses

Two different approaches were used to quantify within-island AFLP diversity. First, the program TFPGA [105] was used to calculate Nei’s [81] unbiased heterozygosity. Allele frequencies were calculated from the dominant AFLP marker data using the allele frequency estimator of Lynch and Milligan [106]. Second, the program MANTEL-STRUCT [107] was used to obtain average pairwise band sharing coefficients between individuals within islands. This procedure calculates the average proportion of shared AFLP marker phenotypes among individuals.

Genetic structuring among islands and island groups was characterized using Wier and Cockerham’s [108] estimate (θ_ST_) of Wright’s F_ST_ in Arlequin v.3.5 [109]. Ninety-five percent confidence limits of θ_ST_ were obtained via bootstrapping over loci. Given that other analyses of our genetic data indicated the presence of two distinct island groups (see Results), we also estimated θ_ST_ and its associated confidence limits for a posteriori grouping of islands. To further refine our inferences about patterns of genetic structure among islands, we used TFPGA to generate a UPGMA dendrogram based on pairwise Nei’s [81] unbiased genetic distances. Support for resulting dendrogram clusters was quantified using a bootstrap procedure over loci (sensu Felsenstein 1985 [110]). To further characterize patterns of genetic differentiation, we used the program NTSYSpc (Exeter Software, Inc.) to perform a principal coordinates ordination to visualize patterns of (dis) similarity among all individuals included in AFLP analyses.

To identify the number of discrete populations (*K*) that occur within the sampled *H. rostratus* we used a Bayesian clustering algorithm implemented in STRUCTURE v.2.3.4 [82]. To assess additional possible substructure, subsets of the data that formed distinct clusters in initial analyses were also subject to the same analyses (southern island group; northern island group; Mahé). Each individual analysis comprised four replicates of 100,000 steps with a 10,000 step burn-in. Given our assumption that individuals from the different islands have not been in contact, the no-admixture model with correlated allele frequencies was used [111]; tested *K* values were specified based on the number of islands for which data were generated for (i.e. *K1* – *K7* for the full dataset) in each analysis. An analysis of *H. rostratus* genetic variation within the largest, highest and geographically most complex Seychelles island of Mahé, which has the most sampled populations (seven) and specimens (*n* = 97), was carried out using the admixture model to investigate intra-island population structure. To infer the most likely *K* for each dataset STRUCTURE HARVESTER [112] was used to determine Δ*K* [113]. The most likely *K* value was then subjected to independent runs on STRUCTURE of 1 × 10^6^ with a burn-in of 100,000 steps. Final summary figures of STRUCTURE results were created using distruct v.1.1 [114].

#### Phenotypic data analyses

Nussbaum & Pfrender [115] presented evidence of sexual size dimorphism (SSD) in Frégate *H. rostratus*. We tested for SSD across all sampled islands using a two-factor analysis of covariance (ANCOVA) with TL as the covariate implemented using PROC GLM [116]. Using ANCOVA, trait means were adjusted for TL and tested for effects of sex, island of origin, and their interaction. Results from this analysis suggested strong dimorphism between males and females for body width and head dimension traits. Subsequent phenotypic multivariate analyses and estimation of levels of population subdivision for phenotypic traits were conducted separately for each sex.

Multivariate analyses for measures and counts of 16 phenotypic traits in 170 males and 146 females were conducted separately for each sex to determine patterns of morphological variation among islands. Principal components analysis (PCA) of metric characters (transformed relative to TL using the allometric vs. standard method [117]) and principal coordinates analysis (PCoA) of metric + meristic variables was implemented using PAST3 [118]. In addition, we estimated Mahalanobis distance matrices using PROC CANDISC (SAS 2003) to generate a matrix of pairwise island phenotypic distances. This matrix was used to construct UPGMA dendrograms of among island morphological variation for each sex using MEGA v.3.1 [119].

To examine patterns of phenotypic divergence on a trait-by-trait basis we obtained restricted Maximum Likelihood (ML) estimates of the variance components explained by island of origin (V_i_) and error (V_e_) for each trait using PROC VARCOMP [116]. Island and error variance estimates are analogous to estimates of the within and among island components of variance, respectively. These estimates were then used to calculate the level of population subdivision for phenotypic characters, P_ST_, using the formula V_i_/(V_i_ + 2(V_e_)). This measure is analogous to the standard measure of quantitative trait subdivision Q_ST_ [120, 121]. However, because our measurements were not taken from individuals reared in a common environment we cannot partition genetic from environmental effects, and thus P_ST_ includes both genetic and environmental sources of variance. We used a nested design, with islands nested within groups (southern, and northern + Frégate island groups) to estimate the levels of subdivision at the level of island group. Because the univariate ANCOVA demonstrated significant sexual dimorphism we examined the correlation between males and females in population subdivision across all phenotypic traits using a least-squared regression of P_ST_ values.

#### Combined data analysis

To assess factors influencing evolution within *H. rostratus,* simple and partial Mantel tests [122, 123] were employed to investigate isolation-by-distance (IBD) and isolation-by-adaptation (IBA), respectively. Morphometric (male and female), molecular (AFLP and mtDNA) and geographic distance data were used in IBD and IBA comparisons to investigate correlations among datasets. The *vegan* package [124] as implemented in R v. 3.2.3 [125] was used for all tests. Analyses were completed using the Pearson method with 999 permutations. For direct comparisons, only islands that had data available across all sampled datasets were used in tests, leaving a total of seven islands: Curieuse, Frégate, La Digue, Mahé, Praslin, Ste. Anne, Silhouette.

To produce distance matrices for the Mantel tests, appropriate analyses for each dataset were completed. For the morphometric data, Gower similarity coefficients for males and females were used and generated by PAST v.3.05 [118]. For AFLPs, Φ_PT_ were generated using 999 permutations with the Microsoft EXCEL add-in GenA1Ex v.6.502 [126, 127]. For mtDNA data the Kimura two-parameter (K2P) model [128] was used to generate K2P distances in MEGA X [97] with partial deletion. The Geographic Distance Matrix Generator v.1.2.3 (Ersts; http://biodiversityinformatics.amnh.org/open_source/gdmg/) was used to generate a pairwise distance matrix for each geographic sampling locality. The probability of the observed correlation was estimated by comparison with a distribution of correlation coefficients generated with 1000 random permutations of matrix elements.

## Supplementary information

**Additional file 1.** Fig. S1. a) UPGMA dendrogram of AFLP data. Support values are represented on the branches, where applicable. b) PCA plot of AFLP data for PCs 1 and 2. Fig. S2. a-e) correlation plots between datasets; f) Morphological trait subdivision (P_ST_*)* among islands. The average level of subdivision among islands for AFLP markers *(θ*_*st*_*)* is denoted by the solid line with the upper and lower confidence limits shown as dotted lines. See text for an explanation of the trait abbreviations and methodology; g-h) male vs. female correlation plots. Fig. S3. Fig. S3. PCA plots for: a) females, b) males. Colors and symbols are the same as those used in Fig. 6. Table S1. Primers used for PCR and Sanger sequencing. Table S2. Morphological patterns of variation between sexes and among islands. Least-squared adjusted means (one SE) and significance values for main effects of sex, island and sex-by-island interaction based on analysis of covariance (ANCOVA) with total length as the covariate using Type III SSR. Table S3. Mahalanobis’ D between islands based on multivariate analysis of 16 morphological characters. See text for an explanation of the analysis. Males (Upper triangle) Females (Lower triangle)

**Additional file 2.** List of specimens used in this study and associated GenBank accession numbers for sequence data.

**Additional file 3.** Results of the final five independent IMa runs.

## Data Availability

All sequence data generated in this study is available on GenBank (cytb = MT569151 – MT569250, *bdnf =* MT569251 – MT569274, *pomc* = MT569275 – MT569293, *brev5* = MT569294 – MT569319, *rost5* = MT569320 – MT569344: see Additional File 2 for full details). Sequence alignments, AFLP data, morphological data, XML files and R scripts are deposited on the NHM Data Portal (10.5519/0002481).

## Abbreviations

MA: Millions of years ago
mtDNA: Mitochondrial DNA
nuDNA: Nuclear DNA
AFLP: Amplified Fragment Length Polymorphism
PCR: Polymerase chain reaction
*cytb*: Cytochrome *b* (gene)
*bdnf*: brain-derived neurotrophic factor (gene)
*pomc*: Pro-opiomelanocortin (gene)
