# Supplementary material for: The roles of vicariance and isolation by distance in shaping biotic diversification across an ancient archipelago: evidence from a Seychelles caecilian amphibian
Source: BMC Evol Biol. 2020 Aug 26;20:110. doi: 10.1186/s12862-020-01673-w (PMC7448330; doi:10.1186/s12862-020-01673-w)
Supplement: Supplementary file 1 — Additional file 1. Fig. S1. a) UPGMA dendrogram of AFLP data. Support values are represented on the branches, where applicable. b) PCA plot of AFLP data for PCs 1 and 2. Fig. S2. a-e) correlation plots between datasets; f) Morphological trait subdivision (PST) among islands. The average level of subdivision among islands for AFLP markers (θst) is denoted by the solid line with the upper and lower confidence limits shown as dotted lines. See text for an explanation of the trait abbreviations and methodology; g-h) male vs. female correlation plots. Fig. S3. Fig. S3. PCA plots for: a) females, b) males. Colors and symbols are the same as those used in Fig. 6. Table S1. Primers used for PCR and Sanger sequencing. Table S2. Morphological patterns of variation between sexes and among islands. Least-squared adjusted means (one SE) and significance values for main effects of sex, island and sex-by-island interaction based on analysis of covariance (ANCOVA) with total length as the covariate using Type III SSR. Table S3. Mahalanobis’ D between islands based on multivariate analysis of 16 morphological characters. See text for an explanation of the analysis. Males (Upper triangle) Females (Lower triangle) [file 12862_2020_1673_MOESM1_ESM.docx]

Title: The role of vicariance and isolation by distance in shaping biotic diversification across an ancient archipelago: evidence from a Seychelles caecilian amphibian

Authors: Simon T. Maddock, Ronald A. Nussbaum, Julia J. Day, Leigh Latta IV, Mark Miller, Debra L. Fisk, Mark Wilkinson, Sara Rocha, David J. Gower and Michael E. Pfrender

Fig. S1. a) UPGMA dendrogram of AFLP data. Support values are represented on the branches, where applicable. b) PCA plot of AFLP data for PC’s 1 and 2.


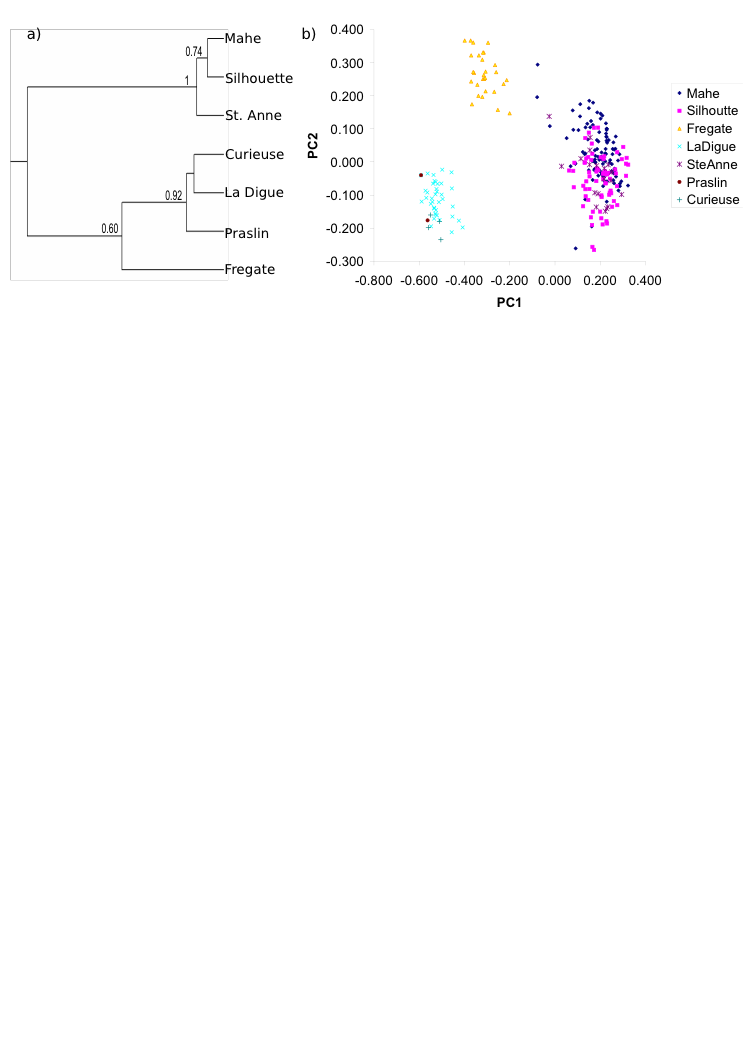


Fig S2. a-e) Correlation plots between datasets; f) Morphological trait subdivision (*P_st_)* among islands. The average level of subdivision among islands for AFLP markers *(θ_st_)* is denoted by the solid line with the upper and lower confidence limits shown as dotted lines. See text for an explanation of the trait abbreviations and methodology; g-h) male vs. female correlation plots.

Fig S3. PCA plots from morphological data for: a) females, b) males. Colors and symbols are the same as those used in Fig 6.

Table S1. Primers used for PCR and Sanger sequencing.

| **Locus** | **Primer name** | **Primer sequence (5’ – 3’)** | **Reference** |
| --- | --- | --- | --- |
| *cytb* | L14910 | GACCTGTGATMTGAAAAACCAYCGTTG | Burbrink et al. (2000) |
|  | H16064 | CTTTGGTTTACAAGAACAATGCTTTA | Burbrink et al. (2000) |
| *pomc* | POMC_DRV_F1 | ATATGTCATGASCCAYTTYCGCTGGAA | Vieites et al. (2007) |
|  | POMV_DRV_R1 | GGCRTTYTTGAAWAGAGTCATTAGWGG | Vieites et al. (2007) |
| *bdnf* | BDNF_DRV_F1 | ACCATCCTTTTCCTKACTATGG | Vieites et al. (2007) |
|  | BDNF_DRV_R1 | CTATCTTCCCCTTTTAATGGTC | Vieites et al. (2007) |
| *brev5* | brev5_F | CATCAGGTCATTGGCGTTTA | Lewis et al. (2014) |
|  | brev5_R | GAGTGCAGGGACCAAATACC | Lewis et al. (2014) |
| *rost5* | rost5_F | TGTCAACTGCCCTCTGTGTC | Lewis et al. (2014) |
|  | rost5_R | AAATTCACAGGCCAAACAGG | Lewis et al. (2014) |

PCR amplification for Sanger sequencing was completed in two time periods and in different labs and are here denoted “*combination 1*” for those amplified in 2003 and “*combination 2*” for those amplified post-2012. Amplifications for *cytb* (*combination 1*) were performed in 50 µl reactions using 2 µl of genomic DNA extraction, 5 µl 10x PCR buffer, 5 µl 8mM dNTP mix, 5 µl of each 2 µM primer, and 0.2 µl Taq DNA polymerase (Perkin Elmer Cetus) and water to final volume. Reactions consisted of an initial denaturation step at 94 °C for 5 minutes, followed by 35 cycles of 92 °C for 45 s, 46 °C for 60 s, and 72 °C for 90 s, and a final extension step at 72 °C for 5 minutes. For *combination 1, cytb* was amplified with the primers outlined in Table S1 and the sequences of single stranded DNA was obtained by using these and the primers L15324 (5'-CCATGAGGACAAATATCATTC -3') and L15584 (5'- TCCCATTYCACCCATACC A-3') (Burbrink *et al.* 2000), and BH3 (5'-GAATGATAYTTCCTATTCGC -3') (Palumbi *et al.* 1991) to generate overlapping fragments of PCR products. Amplifications for *cytb* (*combination 2*) [L14910 & H16064] and nuDNA were performed in 25 µl reactions using 1 µl of genomic DNA extraction, 12.5 µl of MyTaq Mix x 2, 1 µl of each primer [outlined in Table S3), and water to final volume. Negative controls were performed for all reactions. PCR products were purified using spin columns (QIAGEN Inc.). PCR products were sequenced in both forward and reverse directions (using the same primers used for PCR amplification) and assembled into contiguous sequences using SEQUENCHER 4.2.2 (Gene Codes) and Geneious v.6.1.4 (Biomatters). Sequences were aligned using default settings for MUSCLE (Edgar 2004) in Geneious. Sequences were checked for unexpected stop codons and indels in MEGA v.6.0.6 (Tamura *et al.* 2013).

Table S2. Morphological patterns of variation between sexes and among islands. Least-squared adjusted means (one SE) and significance values for main effects of sex, island and sex-by-island interaction based on analysis of covariance (ANCOVA) with total length as the covariate using Type III SSR.

| Trait | Island | Males | Females | Source | DF | F Value | Pr > F |
| --- | --- | --- | --- | --- | --- | --- | --- |
| Body Width | Mahé | 7.93 (0.98) | 8.19 (0.88) | Sex | 1 | 11.99 | 0.0006 |
|  | Silhouette | 8.46 (1.83) | 8.56 (2.34) | Island | 7 | 18.12 | <0.0001 |
|  | Fregate | 9.45 (2.21) | 9.94 (1.99) | Sex*Island | 7 | 1.12 | 0.3464 |
|  | La Digue | 9.04 (1.80) | 9.09 (2.38) | TL | 1 | 1701.72 | <0.0001 |
|  | Ste. Anne | 7.99 (3.31) | 8.59 (3.12) |  |  |  |  |
|  | Praslin | 8.11 (1.55) | 8.15 (1.54) |  |  |  |  |
|  | Curieuse | 7.96 (4.20) | 9.88 (6.58) |  |  |  |  |
|  | Felicite | 8.79 (3.10) | 9.31 (2.81) |  |  |  |  |
|  |  |  |  |  |  |  |  |
| Head Length | Mahé | 7.57 (0.43) | 7.20 (0.38) | Sex | 1 | 16.14 | <0.0001 |
|  | Silhouette | 7.66 (0.79) | 7.31 (1.02) | Island | 7 | 73.47 | <0.0001 |
|  | Fregate | 7.18 (0.96) | 6.90 (0.87) | Sex*Island | 7 | 2.29 | 0.0266 |
|  | La Digue | 6.84 (0.78) | 6.19 (1.03) | TL | 1 | 4031.38 | <0.0001 |
|  | Ste. Anne | 7.04 (1.44) | 7.00 (1.35) |  |  |  |  |
|  | Praslin | 6.41 (0.68) | 6.29 (0.67) |  |  |  |  |
|  | Curieuse | 6.35 (1.83) | 6.42 (2.86) |  |  |  |  |
|  | Felicite | 6.57 (1.35) | 6.32 (1.22) |  |  |  |  |
|  |  |  |  |  |  |  |  |
| Head Width | Mahé | 5.25 (0.35) | 5.05 (0.31) | Sex | 1 | 14.08 | 0.0002 |
|  | Silhouette | 5.09 (0.65) | 5.05 (0.84) | Island | 7 | 75.64 | <0.0001 |
|  | Fregate | 5.23 (0.79) | 4.90 (0.71) | Sex*Island | 7 | 3.29 | 0.002 |
|  | La Digue | 4.62 (0.64) | 4.05 (0.85) | TL | 1 | 3994.13 | <0.0001 |
|  | Ste. Anne | 5.25 (1.18) | 4.92 (1.11) |  |  |  |  |
|  | Praslin | 4.33 (0.56) | 4.27 (0.55) |  |  |  |  |
|  | Curieuse | 4.57 (1.50) | 4.79 (2.35) |  |  |  |  |
|  | Felicite | 4.49 (1.11) | 4.27 (1.00) |  |  |  |  |
|  |  |  |  |  |  |  |  |
| Inter-Ocular Distance | Mahé | 3.60 (0.25) | 3.39 (0.23) | Sex | 1 | 16.49 | <0.0001 |
|  | Silhouette | 3.60 (0.48) | 3.52 (0.61) | Island | 7 | 69.17 | <0.0001 |
|  | Fregate | 3.76 (0.57) | 3.43 (0.52) | Sex*Island | 7 | 3.28 | 0.0021 |
|  | La Digue | 3.24 (0.47) | 2.82 (0.62) | TL | 1 | 4203.87 | <0.0001 |
|  | Ste. Anne | 3.50 (0.86) | 3.32 (0.81) |  |  |  |  |
|  | Praslin | 2.98 (0.40) | 2.90 (0.40) |  |  |  |  |
|  | Curieuse | 3.02 (1.09) | 3.30 (1.71) |  |  |  |  |
|  | Felicite | 3.01 (0.81) | 2.84 (0.73) |  |  |  |  |
|  |  |  |  |  |  |  |  |
| Inter-Naris Distance | Mahé | 1.76 (0.16) | 1.66 (0.15) | Sex | 1 | 22.43 | <0.0001 |
|  | Silhouette | 1.76 (0.31) | 1.68 (0.39) | Island | 7 | 35.2 | <0.0001 |
|  | Fregate | 1.68 (0.37) | 1.50 (0.33) | Sex*Island | 7 | 1.86 | 0.0749 |
|  | La Digue | 1.64 (0.30) | 1.44 (0.40) | TL | 1 | 2183.43 | <0.0001 |
|  | Ste. Anne | 1.62 (0.56) | 1.51 (0.52) |  |  |  |  |
|  | Praslin | 1.40 (0.26) | 1.38 (0.26) |  |  |  |  |
|  | Curieuse | 1.62 (0.71) | 1.48 (1.11) |  |  |  |  |
|  | Felicite | 1.58 (0.52) | 1.52 (0.47) |  |  |  |  |
|  |  |  |  |  |  |  |  |
| Eye-Naris Distance | Mahé | 3.26 (0.23) | 3.05 (0.21) | Sex | 1 | 27.85 | <0.0001 |
|  | Silhouette | 3.24 (0.43) | 3.12 (0.55) | Island | 7 | 85.32 | <0.0001 |
|  | Fregate | 3.07 (0.52) | 2.67 (0.47) | Sex*Island | 7 | 2.66 | 0.0105 |
|  | La Digue | 2.77 (0.42) | 2.47 (0.56) | TL | 1 | 3506.88 | <0.0001 |
|  | Ste. Anne | 3.22 (0.77) | 2.90 (0.73) |  |  |  |  |
|  | Praslin | 2.59 (0.36) | 2.52 (0.36) |  |  |  |  |
|  | Curieuse | 2.50 (0.98) | 2.61 (1.54) |  |  |  |  |
|  | Felicite | 2.71 (0.73) | 2.56 (0.66) |  |  |  |  |
|  |  |  |  |  |  |  |  |
| Eye-Tent Distance | Mahé | 2.44 (0.21) | 2.26 (0.19) | Sex | 1 | 22.86 | <0.0001 |
|  | Silhouette | 2.43 (0.39) | 2.31 (0.50) | Island | 7 | 57.31 | <0.0001 |
|  | Fregate | 2.18 (0.47) | 1.95 (0.43) | Sex*Island | 7 | 2.1 | 0.0421 |
|  | La Digue | 2.11 (0.39) | 1.84 (0.51) | TL | 1 | 2979.3 | <0.0001 |
|  | Ste. Anne | 2.43 (0.71) | 2.13 (0.67) |  |  |  |  |
|  | Praslin | 1.95 (0.33) | 1.88 (0.33) |  |  |  |  |
|  | Curieuse | 1.91 (0.90) | 2.07 (1.42) |  |  |  |  |
|  | Felicite | 1.99 (0.67) | 1.81 (0.60) |  |  |  |  |
|  |  |  |  |  |  |  |  |
| Tent-Nare Distance | Mahé | 1.11 (0.10) | 1.03 (0.09) | Sex | 1 | 6.74 | 0.0097 |
|  | Silhouette | 1.11 (0.19) | 1.08 (0.25) | Island | 7 | 49.89 | <0.0001 |
|  | Fregate | 1.04 (0.23) | 0.92 (0.21) | Sex*Island | 7 | 4.21 | 0.0002 |
|  | La Digue | 0.97 (0.19) | 0.82 (0.25) | TL | 1 | 1782.83 | <0.0001 |
|  | Ste. Anne | 1.01 (0.35) | 0.97 (0.33) |  |  |  |  |
|  | Praslin | 0.85 (0.16) | 0.85 (0.16) |  |  |  |  |
|  | Curieuse | 0.82 (0.44) | 0.94 (0.69) |  |  |  |  |
|  | Felicite | 0.95 (0.33) | 0.94 (0.30) |  |  |  |  |
|  |  |  |  |  |  |  |  |
| Primary Folds | Mahé | 99.47 (0.19) | 100.17 (0.17) | Sex | 1 | 0.34 | 0.5599 |
|  | Silhouette | 97.54 (0.35) | 97.32 (0.45) | Island | 7 | 81.73 | <0.0001 |
|  | Fregate | 95.88 (0.43) | 95.13 (0.39) | Sex*Island | 7 | 1.48 | 0.1724 |
|  | La Digue | 100.98 (0.35) | 100.98 (0.46) | TL | 1 | 0.03 | 0.856 |
|  | Ste. Anne | 99.86 (0.64) | 98.88 (0.60) |  |  |  |  |
|  | Praslin | 102.78 (0.30) | 102.79 (0.30) |  |  |  |  |
|  | Curieuse | 105.38 (0.81) | 104.49 (1.27) |  |  |  |  |
|  | Felicite | 100.89 (0.60) | 101.72 (0.54) |  |  |  |  |
|  |  |  |  |  |  |  |  |
| Ventral Folds | Mahé | 2.22 (0.06) | 2.28 (0.06) | Sex | 1 | 0.99 | 0.3213 |
|  | Silhouette | 1.34 (0.12) | 1.68 (0.15) | Island | 7 | 14.03 | <0.0001 |
|  | Fregate | 2.01 (0.14) | 2.24 (0.13) | Sex*Island | 7 | 1.17 | 0.3193 |
|  | La Digue | 2.08 (0.11) | 2.20 (0.15) | TL | 1 | 0.21 | 0.6486 |
|  | Ste. Anne | 1.38 (0.21) | 1.79 (0.20) |  |  |  |  |
|  | Praslin | 1.91 (0.10) | 1.72 (0.10) |  |  |  |  |
|  | Curieuse | 2.22 (0.27) | 2.01 (0.42) |  |  |  |  |
|  | Felicite | 2.67 (0.20) | 2.64 (0.18) |  |  |  |  |
|  |  |  |  |  |  |  |  |
| Secondary Folds | Mahé | 18.23 (0.65) | 17.82 (0.58) | Sex | 1 | 0 | 0.9983 |
|  | Silhouette | 9.96 (1.20) | 10.04 (1.53) | Island | 7 | 25.34 | <0.0001 |
|  | Fregate | 9.47 (1.44) | 8.70 (1.31) | Sex*Island | 7 | 0.2 | 0.9847 |
|  | La Digue | 11.10 (1.17) | 10.61 (1.56) | TL | 1 | 0.29 | 0.5923 |
|  | Ste. Anne | 14.87 (2.17) | 17.88 (2.04) |  |  |  |  |
|  | Praslin | 10.54 (1.02) | 9.95 (1.01) |  |  |  |  |
|  | Curieuse | 12.21 (2.75) | 11.56 (4.31) |  |  |  |  |
|  | Felicite | 9.58 (2.03) | 9.40 (1.84) |  |  |  |  |
|  |  |  |  |  |  |  |  |
| Complete Secondary Folds | Mahé | 3.92 (0.19) | 3.58 (0.17) | Sex | 1 | 0.22 | 0.6383 |
|  | Silhouette | 2.74 (0.35) | 2.30 (0.45) | Island | 7 | 7.12 | <0.0001 |
|  | Fregate | 2.90 (0.42) | 2.82 (0.39) | Sex*Island | 7 | 0.66 | 0.7038 |
|  | La Digue | 3.06 (0.35) | 3.14 (0.46) | TL | 1 | 2.25 | 0.1347 |
|  | Ste. Anne | 2.47 (0.64) | 3.86 (0.60) |  |  |  |  |
|  | Praslin | 2.19 (0.30) | 2.31 (0.30) |  |  |  |  |
|  | Curieuse | 2.77 (0.81) | 3.05 (1.27) |  |  |  |  |
|  | Felicite | 2.46 (0.60) | 2.48 (0.54) |  |  |  |  |
|  |  |  |  |  |  |  |  |
| Vertebral Count | Mahé | 104.76 (0.22) | 105.73 (0.22) | Sex | 1 | 0.53 | 0.4654 |
|  | Silhouette | 103.92 (0.34) | 103.35 (0.42) | Island | 7 | 69.32 | <0.0001 |
|  | Fregate | 101.00 (0.41) | 100.41 (0.37) | Sex*Island | 7 | 2.42 | 0.02 |
|  | La Digue | 105.63 (0.34) | 105.75 (0.44) | TL | 1 | 0 | 0.9987 |
|  | Ste. Anne | 105.63 (0.61) | 104.33 (0.56) |  |  |  |  |
|  | Praslin | 108.17 (0.36) | 108.39 (0.41) |  |  |  |  |
|  | Curieuse | 110.20 (0.78) | 108.67 (0.99) |  |  |  |  |
|  | Felicite | 105.44 (0.57) | 106.64 (0.52) |  |  |  |  |
|  |  |  |  |  |  |  |  |
| Scale Rows | Mahé | 2.86 (0.10) | 2.80 (0.09) | Sex | 1 | 0.57 | 0.4508 |
|  | Silhouette | 3.22 (0.17) | 2.96 (0.22) | Island | 7 | 7.51 | <0.0001 |
|  | Fregate | 3.48 (0.21) | 3.49 (0.19) | Sex*Island | 7 | 1.17 | 0.321 |
|  | La Digue | 2.91 (0.17) | 3.36 (0.23) | TL | 1 | 48.59 | <0.0001 |
|  | Ste. Anne | 3.79 (0.31) | 3.94 (0.30) |  |  |  |  |
|  | Praslin | 2.41 (0.15) | 2.75 (0.15) |  |  |  |  |
|  | Curieuse | 2.80 (0.40) | 2.40 (0.63) |  |  |  |  |
|  | Felicite | 3.09 (0.29) | 3.69 (0.27) |  |  |  |  |
|  |  |  |  |  |  |  |  |
| Primary Folds with Scales | Mahé | 74.79 (0.99) | 73.77 (0.88) | Sex | 1 | 0.02 | 0.8828 |
|  | Silhouette | 66.17 (1.70) | 62.46 (2.24) | Island | 7 | 7.11 | <0.0001 |
|  | Fregate | 72.49 (2.03) | 74.52 (1.84) | Sex*Island | 7 | 0.43 | 0.8828 |
|  | La Digue | 72.68 (1.67) | 72.61 (2.20) | TL | 1 | 57.03 | <0.0001 |
|  | Ste. Anne | 71.63 (3.06) | 72.61 (2.88) |  |  |  |  |
|  | Praslin | 71.60 (1.45) | 72.19 (1.45) |  |  |  |  |
|  | Curieuse | 76.77 (3.89) | 75.12 (6.09) |  |  |  |  |
|  | Felicite | 77.13 (2.87) | 78.55 (2.59) |  |  |  |  |

| Table S3**.** Mahalanobis’ D between islands based on multivariate analysis of 16 morphological characters. See text for an explanation of the analysis. Males (Upper triangle) Females (Lower triangle) | | | | | | | | |
| --- | --- | --- | --- | --- | --- | --- | --- | --- |
|  |  |  |  |  |  |  |  |  |
|  | Island | | | | | | | |
| Island | Mahé | Silhoutte | Frégate | La Digue | Ste. Anne | Praslin | Curieuse | Felicite |
|  |  |  |  |  |  |  |  |  |
| Mahé | - | 9.48 | 22.20 | 20.36 | 8.40 | 22.25 | 37.09 | 19.31 |
| Silhouette | 8.20 | - | 15.95 | 19.81 | 15.28 | 21.66 | 43.87 | 19.24 |
| Frégate | 26.32 | 18.01 | - | 24.26 | 28.95 | 35.38 | 54.49 | 22.68 |
| La Digue | 17.09 | 26.05 | 31.38 | - | 22.24 | 9.92 | 12.75 | 4.25 |
| Ste. Anne | 4.34 | 8.07 | 19.12 | 19.14 | - | 22.19 | 35.07 | 22.52 |
| Praslin | 17.82 | 25.51 | 40.16 | 11.28 | 22.34 | - | 11.28 | 11.84 |
| Curieuse | 24.32 | 40.07 | 50.30 | 14.67 | 31.26 | 13.09 | - | 16.89 |
| Felicite | 17.15 | 26.18 | 34.09 | 6.27 | 22.08 | 12.58 | 13.65 | - |
|  |  |  |  |  |  |  |  |  |

References

Burbrink F., Lawson R, Slowinski JB (2000) Mitochondrial DNA phylogeography of the polytypic north american rat snake (Elaphe obsoleta): a critiqe of the subspecies oncept. *Evolution*, **54**, 2107–2118.

Edgar RC (2004) MUSCLE: multiple sequence alignment with high accuracy and high throughput. *Nucleic Acids Research*, **32**, 1792–1797.

Lewis CJ, Maddock ST, Day JJ *et al.* (2014) Development of anonymous nuclear markers from Illumina paired-end data for Seychelles caecilian amphibians (Gymnophiona: Indotyphlidae). *Conservation Genetics Resources*, **6**, 289–291.

Palumbi S, Martin A, Romano S *et al.* (1991) *The Simple Fool’s Guide to PCR, Version 2.0*. Special Publication of the Department of Zoology, University of Hawaii, Honolulu.

Tamura K, Stecher G, Peterson D, Filipski A, Kumar S (2013) MEGA6: Molecular Evolutionary Genetics Analysis version 6.0. *Molecular Biology and Evolution*, **30**, 2725–2729.

Vieites DR, Min M-S, Wake DB (2007) Rapid diversification and dispersal during periods of global warming by plethodontid salamanders. *Proceedings of the National Academy of Sciences of the United States of America*, **104**, 19903–7.
